# Autologous Dendritic Cells in Combination With Chemotherapy Restore Responsiveness of T Cells in Breast Cancer Patients: A Single-Arm Phase I/II Trial

**DOI:** 10.3389/fimmu.2021.669965

**Published:** 2021-08-20

**Authors:** David A. Bernal-Estévez, Mauren A. Ortíz Barbosa, Paola Ortíz-Montero, Claudia Cifuentes, Ramiro Sánchez, Carlos A. Parra-López

**Affiliations:** ^1^Immunology and Clinical Oncology Research Group, Fundación Salud de los Andes, Bogotá, Colombia; ^2^Oncology Department, Hospital Universitario Mayor de Méderi, Bogotá, Colombia; ^3^Immunology and Translational Medicine Research Group, Department of Microbiology, Medical School, Universidad Nacional de Colombia, Bogotá, Colombia

**Keywords:** breast cancer, immunotherapy, dendritic cells, neoadjuvant chemotherapy, clinical trial

## Abstract

**Introduction:**

Animal studies and preclinical studies in cancer patients suggest that the induction of immunogenic cell death (ICD) by neoadjuvant chemotherapy with doxorubicin and cyclophosphamide (NAC-AC) recovers the functional performance of the immune system. This could favor immunotherapy schemes such as the administration of antigen-free autologous dendritic cells (DCs) in combination with NAC-AC to profit as cryptic vaccine immunogenicity of treated tumors.

**Objective:**

To explore the safety and immunogenicity of autologous antigen-free DCs administered to breast cancer patients (BCPs) in combination with NAC-AC.

**Materials and Methods:**

A phase I/II cohort clinical trial was performed with 20 BCPs treated with NAC-AC [nine who received DCs and 11 who did not (control group)]. The occurrence of adverse effects and the functional performance of lymphocytes from BCPs before and after four cycles of NAC-AC receiving DCs or not were assessed using flow cytometry and compared with that from healthy donors (HDs). Flow cytometry analysis using manual and automated algorithms led us to examine functional performance and frequency of different lymphocyte compartments in response to a stimulus *in vitro*. This study was registered at clinicaltrials.gov (NCT03450044).

**Results:**

No grade II or higher adverse effects were observed associated with the transfer of DCs to patients during NAC-AC. Interestingly, in response to the *in vitro* stimulation, deficient phosphorylation of Zap70 and AKT proteins observed before chemotherapy in most patients’ CD4 T cells significantly recovered after NAC-AC only in patients who received DCs.

**Conclusions:**

The transfer of autologous DCs in combination with NAC-AC in BCPs is a safe procedure. That, in BCPs, the administration of DCs in combination with NAC-AC favors the recovery of the functional capacity of T cells suggests that this combination may potentiate the adjuvant effect of ICD induced by NAC-AC on T cells and, hence, potentiate the immunogenicity of tumors as cryptic vaccines.

## Introduction

The development of immunotherapy for patients with breast cancer has been limited by the low immunogenicity of tumor antigens; however, recent studies combining chemotherapy with anti-checkpoint antibodies such as programmed cell death protein 1 (PD-1) and cytotoxic T lymphocyte-associated protein 4 (CTLA-4) (1–4) show that this combination induces better treatment efficacy against solid tumors, including breast cancer. The concept of anthracycline-induced immunogenic cell death (ICD) initially tested in murine models (5) and more recently confirmed in cancer patients (6, 7) and infectious diseases (8) suggests that the combined use of chemotherapy regimens that induce ICD [such as neoadjuvant chemotherapy with doxorubicin and cyclophosphamide (NAC-AC)], in combination with immunotherapy, could be advantageous.

The results recently published by others and preclinical studies performed by our group have shown that the functional deficiency of T cells, dendritic cells (DCs), natural killer (NK) cells, and other immune cells observed in breast cancer patients (BCPs) with invasive ductal carcinoma before treatment is efficiently recovered after NAC-AC (four doses of AC spaced every 3 weeks followed by paclitaxel alone or in combination with trastuzumab and surgery) (8–12). However, NAC-AC have not yet been shown *in vivo* to rescue the unresponsiveness of T cells seen in cancer patients prior to treatment. If this turns out to be the case, tumor antigens and danger signals elicited by tumors treated with NAC-AC could enhance the immunogenicity of the tumors; hence, administration of DC to patients in NAC-AC might promote cross-presentation to activated T cells of these antigens, making the patient’s tumor antigens—whose identification is laborious and its production under Good Manufacturing Practice (GMP) conditions very costly—dispensable for the developing DC-based therapeutic cancer vaccines.

Monocyte-derived DCs pulsed with tumor antigens have been used as natural adjuvants and for the presentation of tumor antigens to T cells in multiple clinical studies, showing a broad spectrum of clinical responses (13). The possibility that NAC-AC *in vivo* favors the induction of ICD and the release of danger signals and tumor antigens from tumor cells led us to hypothesize that the administration of autologous DCs to BCPs receiving NAC-AC will selectively rescue the functional performance of T cells in these patients. To prove this, autologous monocytes were administered in combination with the NAC-AC scheme to a group of BCPs with invasive ductal carcinoma.

In the present phase I/II clinical study, we evaluated the safety and immunogenicity of two types of monocyte-derived DC administered in combination with NAC-AC to patients with invasive ductal breast cancer. The results presented here confirm the safety of the use of these cells in patients with breast cancer under NAC-AC. Using a manual and unbiased analysis system to characterize lymphocyte subpopulations by flow cytometry and the evaluation of the T-cell receptor (TCR) repertoire in a vaccinated patient suggest that the combined use of DCs with NAC-AC increases the functionality of T lymphocytes to levels comparable to cells obtained from healthy donors (HDs).

## Materials and Methods

### Study Design

**Figure 1:**
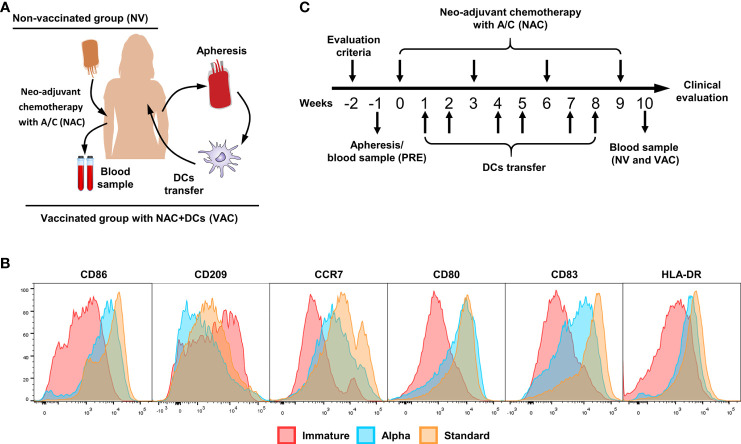
Vaccination scheme and dendritic cell (DC) characterization. **(A)** Patient interventions, before NAC, patients have gone to an apheresis collection to obtain peripheral blood mononuclear cells (PBMCs) and therefore induce monocyte-derived DCs (cryopreserved until use). After four doses of chemotherapy, a new blood sample was taken to compare between pre- and post-chemotherapy. **(B)** Representative histograms of immature DCs (red), alpha DCs (blue), and standard DCs (yellow), comparing the expression levels of CD86, CD209, CCR7, CD80, CD83, and HLA-DR. **(C)** Clinical trial scheme per patient in weeks, week 0 is referred to the first dose of chemotherapy, the process starts with the evaluation criteria for patients (w-2), apheresis (vaccinated group) or blood sample (control group) was taken in w-1. Between doses of chemotherapy, we transferred two doses of DCs for a total of six doses. One week after the fourth dose of A/C, we collect the second blood sample.

This randomized clinical trial was approved by the Universidad Nacional de Colombia Institutional Review Board (Ethics committee No. 016-233-17) and ethics committee of the Universidad del Rosario–Méderi Clinic. This study was registered at www.clinicaltrials.gov (NCT03450044). All patients signed an informed consent, and inclusion criteria were evaluated by the clinical committee. Patients were randomized to one of two groups: (i) patients who received only NAC [non-vaccinated (NV) group] or (ii) patients who received NAC in addition to autologous monocyte-derived DCs [vaccinated (VAC) group] (**Figure 1A**). The primary goal of this clinical trial was to determine the safety of the transfer of autologous DCs during chemotherapy and to assess the immune response generated after six doses of DCs.

### Volunteers and Patient Blood Samples

Samples from HDs and BCPs of the NV group (NAC only) were collected and processed as previously described (10). Briefly, buffy coats were obtained from HDs and BCPs with ductal invasive carcinoma before and after 3 months of chemotherapy with doxorubicin and cyclophosphamide (A/C scheme). Peripheral blood mononuclear cells (PBMCs) were purified using density gradient centrifugation with Ficoll-Paque (GE Healthcare) and cryopreserved in liquid nitrogen in freezing medium containing 50% RPMI + 40% fetal bovine serum (FBS) and 10% dimethyl sulfoxide (DMSO). For samples obtained from patients in the vaccinated group (NAC plus DCs), between 4 and 6 days prior to the first dose of NAC, a leukapheresis was taken (MCS+ 9000; Haemonetics^®^) configured to enrich monocytes and peripheral blood stem cell (PBSC) set (Ref 0971E-00; Haemonetics^®^). The whole leukapheresis was washed several times to remove ACD-A anticoagulant, and PBMCs were enriched using density gradient centrifugation with Ficoll-Paque and freshly used for monocyte enrichment (see details below). The clinical characteristics of the patients involved in this study are summarized in **Table 1**.

**Table 1:** Patients’ clinical characteristics.

Characteristic	NV (n = 11)	VAC (n = 9)
Mean age (years)	54.5	49.1
TNM		
1	2	1
2	8	7
3	1	1
Clinical stage		
II	4	3
III	8	6
Clinical lymph node classification		
N0	2	2
N1	6	5
N2	3	2
N3	0	0
Systemic metastases		
No	11	9
Yes	0	0
Estrogen receptor (ER)		
Positive (>10%)	7	5
Negative (<10%)	4	4
Progesterone receptor (PR)		
Positive (>10%)	7	5
Negative (<10%)	4	4
Ki-67 (%)	42	37
Her2/neu		
Positive	3	2
Negative	8	7
Breast cancer subtypes		
Luminal A	2	1
Luminal B	8	6
Basal like	0	1
Her2/neu overexpressing	1	1

### Monocyte-Derived Dendritic Cell Differentiation

All cell processes were done in a Good Laboratory Practice (GLP)-compliant laboratory at Fundación Salud de los Andes. Monocytes were enriched by plastic adherence of freshly isolated PBMCs for 2 h at 37°C; non-adherent cells were cryopreserved as described before for further use. Adherent cells were differentiated to immature DCs (iDCs) culturing in serum-free medium (AIM-V CST; Thermo Fisher) by adding granulocyte–macrophage colony-stimulating factor (GM-CSF) and interleukin (IL)-4 (GMP grade; CellGenix, Germany) for 36 h and matured using two different cocktails (standard DCs and alpha DCs) of pro-inflammatory cytokines as previously described (14–16). The phenotypes of standard and alpha DCs were confirmed by flow cytometry, staining the cells with fluorescent labeled antibodies against CD80, CD83, CCR7, human leukocyte antigen (HLA)-DR, CD209, and CD86 (all from Biolegend) and compared with iDCs (**Figure 1B**). All mature DCs were cryopreserved in liquid nitrogen until use with autologous serum (40%), AIM-V CTS (50%), and DMSO (10%). Quality control of DCs includes viability, maturation phenotype (**Figure 1B**), and contamination control including bacterial growth, mycoplasma presence by PCR (MycoSEQ™ Mycoplasma Detection Kit; Applied Biosystems™), and endotoxin levels (gel clot determined by an external laboratory). For DC transfer, cryopreserved cells were washed twice for DMSO removal and resuspended in 200 µl of autologous serum.

### Vaccination Scheme and Clinical Monitoring

After informed consent was signed, patients were selected, and the inclusion and exclusion criteria were confirmed. The patients were randomly assigned to receive concomitant with NAC autologous DCs or only NAC. The vaccination schedule of patients with DCs consisted of ~2 × 10^7^ cells per dose for a total of six doses. Between each session of A/C chemotherapy (scheduled every 21 days), we apply two doses of cells, one per week. The cells were injected intradermally near the tumor lesson in the affected breast. During each dose, patients were monitored in the hospital for adverse effects for 3–4 h; after that, a daily check was done to evaluate the appearance of adverse effects (**Figure 1C**).

### Immune Monitoring Evaluation

For functional analysis of T cells, patients’ PBMCs before and after treatment were stimulated with antiCD3/CD2/CD28 beads (Miltenyi Biotec) for 48 h in a ratio 2:1 (cells:beads) in a high-density system (17). After incubation, T-cell activation phenotype was analyzed by flow cytometry using different combinations of the following antibodies: TCR α/β fluorescein isothiocyanate (FITC; Thermo Scientific), CCR7 PE, CD8 PE/Dazzle 594 (Biolegend), phospho-mammalian target of rapamycin (mTOR) (p-mTOR) PerCP eFluor 710 (Ser2448; Thermo Scientific), p-AKT PE-Cy7 (Ser473; Thermo Scientific), p-ZAP70 AF 647 [(Tyr319)/Syk Phospho (Tyr352); Biolegend], CD69 Allophycocyanin (APC)-Cy7 (Biolegend), CD25 Alexa Fluor 700 (Biolegend), CD3 Brilliant Violet 510 (Biolegend), and CD45RO Pacific Blue (Biolegend). CD4 population was defined by CD3-positive CD8-negative lymphocytes. The staining, fixation, and permeabilization processes were done using IntraStain kit as described by the manufacturer (Agilent Dako).

### Flow Cytometry Data Acquisition and Analysis

Data were acquired using FACS Aria II (BD) with a minimum of 10^5^ events in singlets region determined by FSC-A *vs.* FSC-H and SSC-H *vs.* SSC-W gates. Data files were exported in FCS version 3.0. Manual analysis was done with FlowJo v10 (BD). For automated analysis, live/single cells lymphocytes (determined by SSC-A *vs.* FSC-A) were exported to a new FCS file. Automated analysis was done following the strategy described in **Figure S1**. Exported FCS files were analyzed, concatenated, and dimension reduction process was done by tSNE with a clustering algorithm using FlowSOM v 2.6 (18) in FlowJo followed by a comparison of each sample in the concatenated file to identify the proportions of each cluster and their corresponding phenotype. Finally, we use the CITRUS (19) algorithm implemented in R software (v3.6.3) (20) to identify statistical differences between patient groups of clustered populations *ex vivo* and after *in vitro* stimulation.

### T-Cell Receptor CDR3 Sequence

From one vaccinated patient we obtained a sample from remaining tumor tissue and a draining lymph node after NAC plus DCs, during surgery. Those samples were processed immediately for single-cell suspension and cryopreserved as previously described. DNA was extracted from PBMCs before (PRE) and after NAC plus DC (VAC) treatment (10^7^ PBMCs) simultaneously with cryopreserved cells from tumor (2 × 10^6^ viable cells) and lymph node (10^7^ viable cells) using DNeasy^®^ Blood and tissue DNA extraction kit (Qiagen, Germany) following the manufacturer’s protocol. TCR-Vβ CDR3 regions were then sequenced by ImmunoSEQ technology (Adaptive Biotechnologies, Seattle, WA, USA).

### Statistical Analysis

T-cell response data were normalized based on *in vitro* stimulated cell culture related to their corresponding unstimulated controls for TCR/CD3 internalization [delta of CD3 median fluorescent intensity (MFI) between control and stimulated T cells]. For the evaluation of protein phosphorylation, MFI values of p-ZAP70, p-AKT, and p-mTOR were normalized by fold increase between stimulated and control samples. Non-parametric tests were used to determine statistical differences between groups. Non-parametric Mann–Whitney test between populations was used. Analyses were done with Prism v9 software (GraphPad). For CITRUS analyses, data were processed in R software (v3.6.3) with CITRUS package (v0.08) (19). The Morisita–Horn index of dispersion (or Morisita overlap index) was used to measure the similarity of the sequences for the TCR-CDR3 region using a scale value from 0 (no similarity) to 1 (complete similarity).

## Results

### Transfer of Autologous Monocyte-Derived Dendritic Cells in Combination With Doxorubicin and Cyclophosphamide Is Safe

It is well known that immunotherapy with autologous DCs used in diverse immunotherapy regimes is well tolerated (21). However, to our knowledge, this is the first time that autologous DCs pulsed without any antigen are used in combination with NAC-AC in BCPs. In search of any adverse effects or symptoms related to the transfer of DCs to cancer patients under NAC-AC, patients were closely monitored within a time frame of 4 hours right after vaccination and thereafter every 24 h (the first week) and twice during the year after the DC administration. We detected neither severe adverse effects nor alteration of O_2_ saturation, ECG, and arterial pressure soon after the inoculation of the cells (**Table 2**) nor abnormal deviation of clinical laboratory parameters such as hemogram, coagulation time, and hepatic and renal function in the long-term follow-up (data not shown). Despite that no severe adverse effects were observed—following the fifth and last dose—8–10 h after injecting the DCs, we did detect in most patients (8/9) a skin redness patch limited to the site of the injection that resolved spontaneously 16–24 h later without the need for any specific treatment.

**Table 2:** Frequency of adverse effects by grade in vaccinated patients.

Adverse event	Frequency (n)
Grade 0	12.5% (1)
Grade 1	87.5% (8)
Grade 2	0% (0)
Grade 3	0% (0)
Grade 4	0% (0)
Grade 5	0% (0)

### Lymphocyte Unresponsiveness in Breast Cancer Patients Is Selectively Recovered by the Combined Administration of Doxorubicin and Cyclophosphamide and Dendritic Cells

In a previous study, we evaluated the functional capacity of the T cell and antigen-presenting cells (APC) compartments in BCPs before and after three doses of NAC-AC. We found that before treatment, T cells and DCs exhibited marked unresponsiveness to the *in vitro* stimulation; however, after the treatment, the responsiveness was partially recovered, and this recovery did correlate with patients’ residual cancer burden (10). Herein, we report the results of a phase I/II clinical study conducted to assess the effect in the functional performance of T lymphocytes of BCPs after six doses of autologous monocyte-derived DCs administered during the first three cycles of NAC-AC.

**Figure 2:**
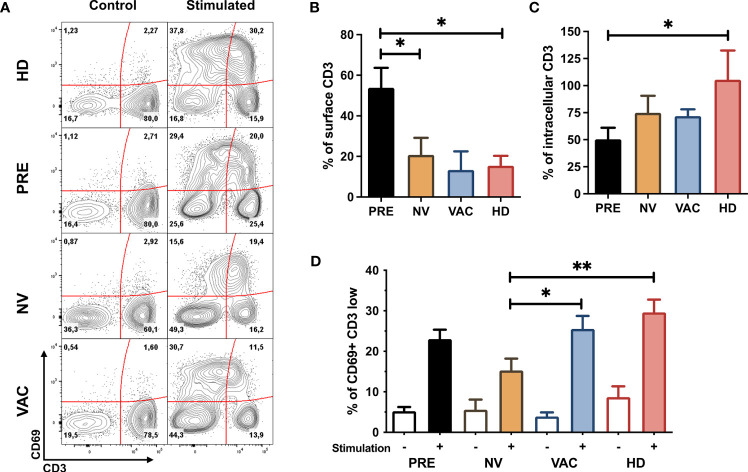
Restoration of T-cell early activation profile after neoadjuvant chemotherapy with A/C (NAC) + dendritic cell (DC) therapy. **(A)** Representative contour plots of CD69 *vs.* CD3 before and after *in vitro* stimulation of peripheral blood mononuclear cells (PBMCs) obtained from healthy donors (HDs) and patients before (PRE) and post-chemotherapy alone [non-vaccinated (NV)] and vaccinated patients (VAC), numbers represent percentage of cells in each quadrant. **(B)** Mean expression of surface CD3 (normalized compared to control samples and represented over a 100%) in HDs (red bar), before chemotherapy (PRE, black bar), non-vaccinated (NV, yellow bar), and vaccinated (VAC, blue bar) patients. **(C)** Quantification of intracellular CD3 (normalized to control samples and represented over a 100%) in the four groups described in panel **(B)**. **(D)** percentage of CD69+CD3low cells in the four groups of samples before stimulation (empty bars) and after *in vitro* stimulation (tinted bars). Non-parametric Mann–Whitney test between populations or groups. *p < 0.05, **p < 0.01.

The responsiveness of lymphocytes especially T cells was determined by flow cytometry evaluating the expression of CD69 (an early activation marker) and the internalization of the TCR/CD3 complex (determined by the relative reduction in CD3 MFI) in cells obtained from the four groups of samples before and after *in vitro* stimulation (**Figure 2A**).

To determine if CD3 molecules were internalized or degraded, we quantified the MFI of internalized CD3 by intracellular staining. We sought to assess the proportion of CD3 at the cell membrane (**Figure 2B**) and in the cytosol (**Figure 2C**) between non-stimulated and stimulated T cells and found that whereas the majority of the CD3 molecules in patients’ T cells tend to degrade, in healthy individuals, T cells tend to accumulate CD3 intracellularly.

T cells from patients who received NAC-AC alone (NV) or in combination with DCs (VAC) internalized the majority of TCR/CD3 complexes in cell surface after *in vitro* stimulation at similar levels of cells obtained from HDs and at a higher proportion compared to samples before chemotherapy (PRE) (**Figures 2B, C**). In contrast, after *in vitro* stimulation, T cells from VAC patients have a higher frequency of CD69 in T cells that have reduced the amount of TCR/CD3 complexes (reduction of CD3 MFI) similar to the response of HD T cells (**Figure 2D**) and in a higher proportion to patients who did not receive DCs (NV).

### Breast Cancer Patients Exhibit T-Cell Signaling Impairment That Recovers After the Administration of Dendritic Cells

**Figure 3:**
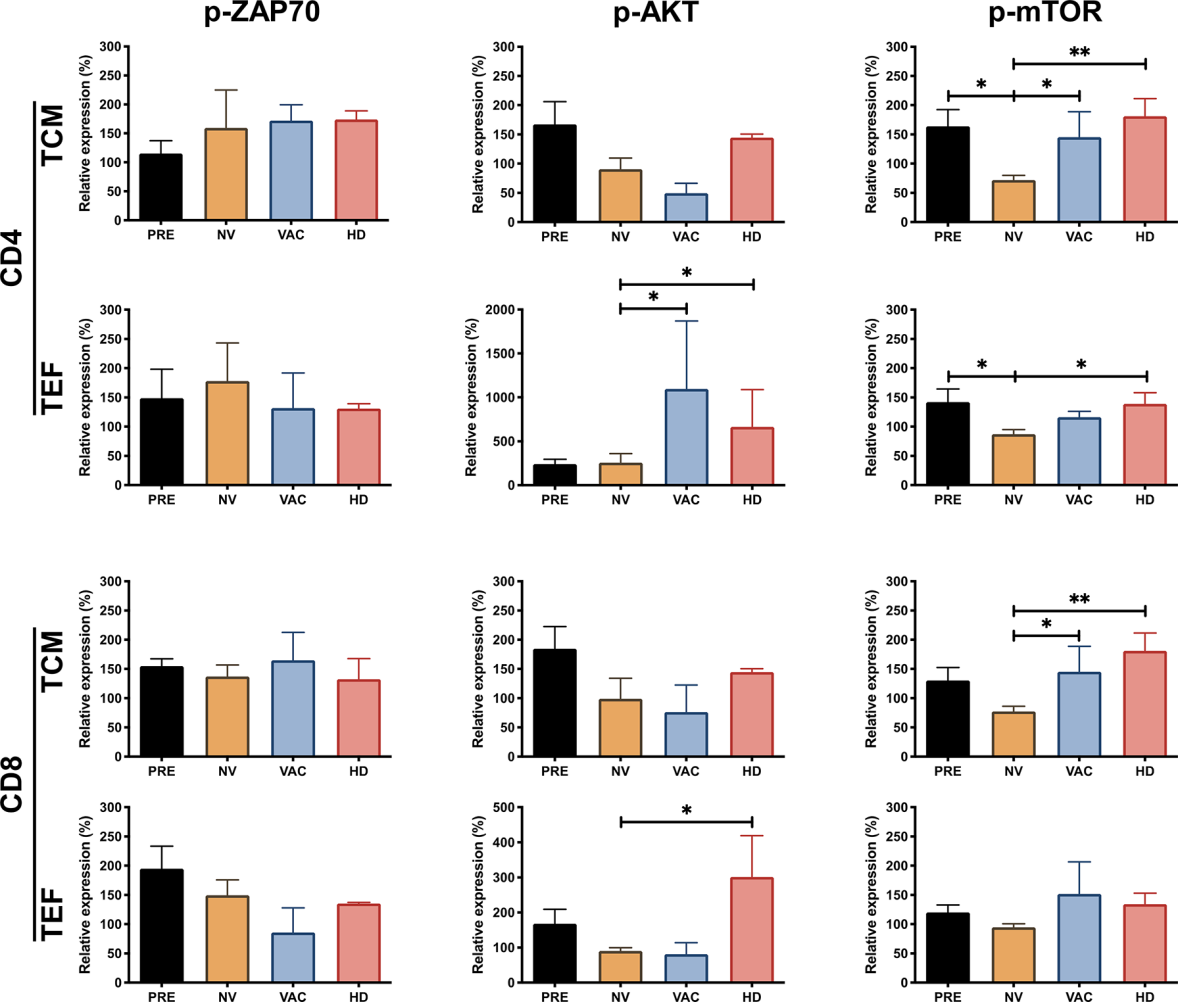
NAC plus dendritic cell (DC) therapy restores T-cell function reflected by the increasing phosphorylation of ZAP70, AKT, and mammalian target of rapamycin (mTOR). Relative expression of p-ZAP70, p-AKT, and p-mTOR in CD4+ and CD8+ T cells, central memory T cells (TCM), and terminal effector T cells (TEF). The expression was normalized based on the median fluorescent intensity (MFI) of each molecule in non-stimulated peripheral blood mononuclear cells (PBMCs) over stimulated cells (represented over 100%) in the four groups, patients before chemotherapy (PRE, black bars), post-chemotherapy alone [non-vaccinated (NV), yellow bars], vaccinated patients (VAC, blue bars), and healthy donors (HDs, red bars). Non-parametric Mann–Whitney test between populations or groups. *p < 0.05, **p < 0.01.

To investigate the molecular defect in the TCR internalization observed in BCPs, we decided to monitor key events of the TCR signaling on T cells. We evaluated by phospho-flow cytometry the activation status of ZAP70, AKT, and mTOR (p-ZAP70, p-AKT, and p-mTOR in **Figure 3**) in central memory (TCM) and effector (TEF) CD4 and CD8 T cells responding to *in vitro* TCR stimulation. We found that before treatment in both CD4 and CD8 T cells (TCM and TEF cells), the proportion of p-ZAP70 among the four groups did not change significantly upon *in vitro* stimulation (**Figure 3**, left column). Interestingly, the expression of p-AKT in the subpopulations analyzed revealed that whereas CD4 and CD8 TEF cells from patients who received chemotherapy (NV or VAC) exhibited a decrease of p-AKT compared to its detection in TEF CD4 T cells in healthy volunteers (HDs), both vaccinated patients (VAC) and HDs exhibited significantly higher p-AKT expression compared to its expression on T cells of unvaccinated individuals (**Figure 3**, middle panel). Furthermore, we observed that p-mTOR was reduced in CD4 and CD8 T cell subpopulations of patients after chemotherapy; however, in patients who received DCs in combination with chemotherapy (VAC), this response was restored to the levels observed in CD4 and CD8 TCM subpopulations of HDs (**Figure 3** right column). Altogether, these results let us to argue in favor of three main findings regarding T-cell activation behaviors observed in the groups of individuals analyzed: (i) the phosphorylation of ZAP70 in T cell subpopulations does not show any significant difference among groups; (ii) NAC-AC affects mTOR and to a lesser extent AKT phosphorylation; and (iii) in patients receiving NAC-AC, DCs restore in activated T cells levels of p-mTOR similar to those observed in HDs.

### Unsupervised Clustering Analysis Identifies Expansion of T-Cell Populations Associated With the Transfer of Dendritic Cells to Patients Under Doxorubicin and Cyclophosphamide

**Figure 4:**
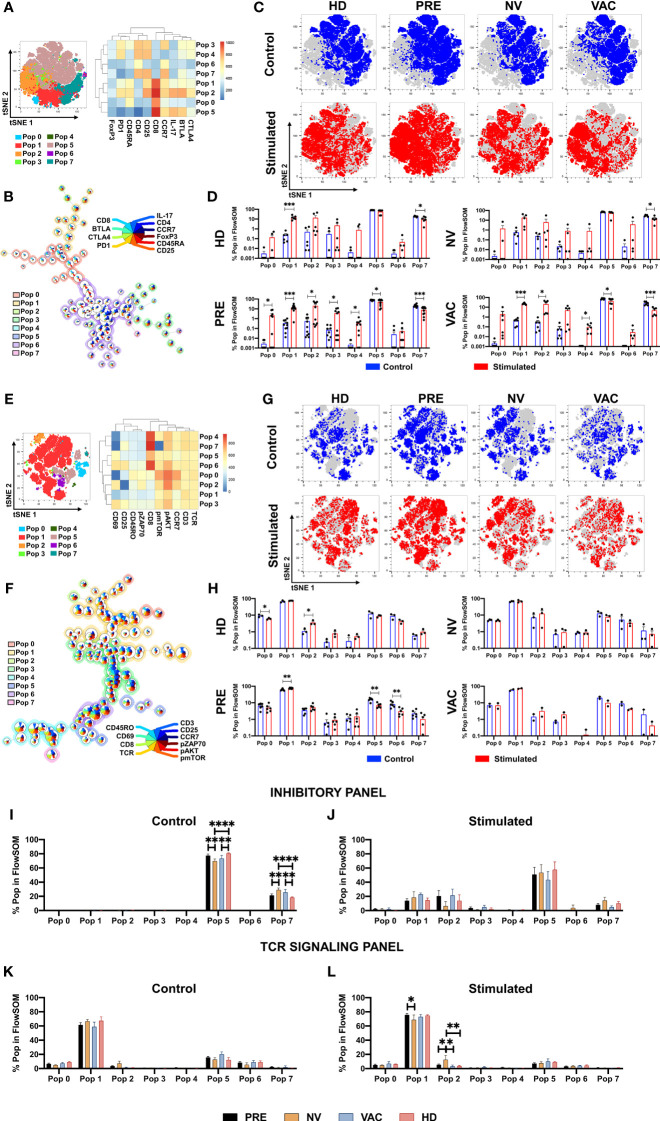
Identification of T-cell populations associated with dendritic cell (DC) transfer by FlowSOM. FlowSOM analysis of two different staining panels, inhibitory **(A**–**D)** and T-cell receptor (TCR) activation **(E**–**H)**. tSNE plots of concatenated samples with the relative location of the eight populations determined by FlowSOM with their respective heat map of each marker **(A**, **E)**. Spanning tree of the FlowSOM populations with their respective pie chart (size of each circle is proportional to cell frequency) **(B**, **F)**. Distribution of stimulated (red) and control (blue) samples in tSNE plot of each patient group **(C**, **G)**. Relative cell frequency in each population for the individual groups between stimulated (red bars) *vs.* control (blue bars) **(D**, **H)**. Direct comparison of the relative cell frequency among groups for the FlowSOM analysis in inhibitory panel (**I**, control; **J,** stimulated) and in the TCR panel (**K**, control; **L**, stimulated). Non-parametric Mann–Whitney test between populations or groups. *p < 0.05, **p < 0.01, ***p < 0.001, and ****p < 0.0001.

To identify characteristics of T cell subpopulations associated with the administration of DCs to cancer patients under NAC-AC, the flow cytometry data of T cells responding to *in vitro* stimulation were analyzed using Pehnograph (data not shown) and FlowSOM (18, 22) algorithm for unsupervised analyses of multiparametric flow cytometry data implemented in FlowJo (BD). This algorithm was tested measuring the internalization of CD3 and the expression of CD25, CD69, and CD154 in samples from HDs after *in vitro* TCR stimulation (**Figures S2A–E**). This analysis evidences a series of clusters of cells that co-express simultaneously the markers used to phenotype the cells facilitating the comparison of clusters among all groups of samples analyzed. As shown in **Figures 4A, E**, FlowSOM identified eight main clusters in a tSNE map based on the expression of two different staining panels, the first panel (inhibitory panel, **Figure 4A**), consisting of antibodies to evaluate the expression of CD4, CD8, CD45RA, CCR7, FoxP3, CD25, B- and T-lymphocyte attenuator (BTLA), CTLA4, and programmed cell death 1 (PD-1) cell markers, and the second panel (activation panel, **Figure 4E**), which evaluates the expression of CD3, CD8, TCRαβ, CD45RO, CCR7, CD69, pZAP70, pAKT, and p-mTOR.

For the inhibitory panel, the expression profile and the hierarchical relation of the eight cell clusters revealed by the FlowSOM analysis represented in the heat map shown in **Figure 4A**, together with their frequency and distribution depicted in the tree scheme presented in **Figure 4B**, led us to conclude that—proportional to circle size—clusters 1, 2, 5, and 7 were the most frequent in the samples analyzed. We then compared inside the tSNE map of cells from the four groups of individuals the cluster distribution of CD8 T cells in cell samples stimulated *vs.* non-stimulated *in vitro* with antiCD3/CD2/CD28 beads (**Figures 4C, D**). Upon *in vitro* stimulation of the cells, it was observed in cells from all groups of individuals except in cells of patients after NAC-AC who did not receive DCs (NV group) the enrichment of populations 1 and 2 (lower left corner in the tSNE map; **Figures 4C, D**). It should be noted that the cluster distribution before and after *in vitro* stimulation was similar in cells of patients before NAC-AC and in patients after the administration of NAC-AC in combination with DC (Pre and VAC groups, respectively). That this distribution was observed neither in cells of patients who received only NAC-AC without DC (NV group) nor in the cells of the HD group is intriguing.

The analyses with FlowSOM of cells stained with the panel designed to assess TCR signaling (**Figures 4E, F**) showed that out the eight populations, population 1 with a CD4 T-cell phenotype (TCR+/CD3+/CD8-) represented the majority of the cells. Those cells distribute differently when stimulated *in vitro* with a significant increase in HDs of population 2 (**Figures 4G, H**), a population of CD4 T cells expressing pAKT and pZAP70. Results presented in **Figure 4H** also show that NAC-AC restitutes the activation and expansion of population 6 (a CD8 T-cell population that upregulates CD69, CD25, pAKT, and pmTOR upon TCR stimulation *in vitro*).

We then sought differences in populations’ percentage between the four groups of individuals studied. Using the inhibitory panel (**Figures 4I, J**), we observed in HDs compared to NV and VAC patients after NAC-AC a significant increase *ex vivo* of population 5 [a CD8+ T-cell population characterized by a central memory phenotype that expresses IL-17 and BTLA (**Figure 4I**)]; notably, these differences were not sustained when the cells were stimulated *in vitro* (**Figure 4J**). Likewise, the increase *ex vivo* of population 7 (an effector CD4+ T-cell population CD25+ FoxP3-) observed in patients after NAC-AC when compared to HD was lost when the cells were stimulated *in vitro*. Finally, when the TCR activation panel was used (**Figures 4K, L**), only the cells of NV patients stimulated *in vitro* (but not *ex vivo*) exhibited the increased expansion of population 2 (an activated CD4 T-cell population CCR7+ characterized by high expression of CD69, pAKT, p-mTOR, and pZAP70).

### The Administration of Autologous Dendritic Cells to Patients With Doxorubicin and Cyclophosphamide Selectively Favors the Expansion and Activation of T Cells

To our knowledge, CITRUS is the only algorithm available for unsupervised analysis of flow cytometry data, which includes statistical analyses that allow distinguishing differences between groups (19). Therefore, CITRUS was used to compare in patients with breast cancer under NAC-AC who received or not autologous DCs the expansion of defined subpopulations of T cells (**Figures S2F–I**). Therefore, cells from the four groups of individuals *ex vivo* and *in vitro* after stimulation of the cells with anti-CD3/CD2/CD28 beads were stained with a panel of antibodies fluorescently labeled for CD3, CD8, CD45RO, CD62L, CD69, and CD25, and through unsupervised analysis, CITRUS selected and analyzed in the four groups of individuals differences or commonalities and phenotype on T-cell subpopulations of interest.

**Figure 5:**
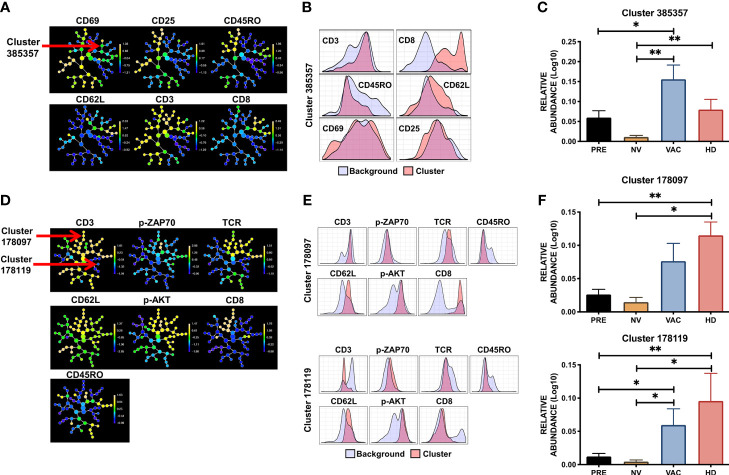
Specific population of T cells are favored by combination therapy NAC + dendritic cells (DCs). CITRUS analysis of peripheral blood mononuclear cell (PBMC) samples obtained from healthy donors (HDs), before chemotherapy (PRE), non-vaccinated (NV), and vaccinated (VAC) patients based on the T-cell receptor (TCR) signaling FC panel. **(A)** Cluster distribution (hierarchy) and expression level of the corresponding marker from low (blue) to high expression (yellow) for each marker, arrow points at cluster 385357. **(B)** Histograms depicting the phenotype of cluster 385357 (red histograms) relative to background expression (blue histograms) for each marker (CD3, CD8, CD45RO, CD62L, CD25, and CD69). **(C)** Relative abundance (Log_10_) of cluster 385357 in the four groups, patients before chemotherapy (PRE, black bars), post-chemotherapy alone [non-vaccinated (NV), yellow bars], vaccinated patients (VAC, blue bars), HDs (red bars). **(D)** Cluster hierarchy and expression levels of TCR signaling panel in e*x vivo* samples, arrows point to clusters 178097 and 178119. **(E)** Histograms of clusters 178097 and 178119 (red histograms) compared to background expression (blue histograms) in each cluster. **(F)** Relative expression (Log_10_) of clusters 178097 and 178119. Non-parametric Mann–Whitney test between populations or groups. *p < 0.05, **p < 0.01.

CITRUS generates a clustering tree in which the expression of each marker within each cluster is in a relative Log_10_ abundance scale (**Figure 5A**). The use of CITRUS evidenced cluster 385357 characterized by phenotype CD8/CD62-L high, CD3/CD69 dim, and CD45RO/CD25 low (red *vs.* blue histograms in **Figure 5B**) whose relative level of detection was statistically different among the four groups (**Figure 5C**). Notably, in patients who received NAC-AC, this naive-like CD8+ T-cell population with intermediate levels of activation that was significantly absent in patients who did not receive DCs (NV) was highly detected in patients who did receive DCs (VAC) (**Figure 5C**). Using the second panel of antibodies that includes p-ZAP70, p-AKT, TCR, CD3, CD45RO, CD62-L, and CD8, CITRUS defined two cell clusters, 178097 and 178119 (**Figures 5D, E**), that were significantly overexpressed in cell samples from VAC patients and HD volunteers (**Figure 5F**). Cluster 178097 has a phenotype of naive-like CD8+ T cells (CD3+/CD8+/CD62-L+/CD45RO-) (**Figure 5E** top), cluster 178119 has an NK-like cell phenotype (CD3-/TCR-/CD8-/CD45RO-/CD62L+) (**Figure 5E** bottom), and both clusters exhibit increased levels of p-AKT and p-ZAP70. Notably, whereas both clusters were relatively underexpressed in patients before NAC-AC and in patients after NAC-AC but who did not receive DCs (PRE and NV, respectively), they were highly expressed in patients who received DCs and in healthy control groups (VAC and HDs in **Figure 5F**). Taking together the results using unsupervised algorithms to analyze T-cell activation and signaling in response in an *in vitro* stimulation, our results lead us to argue that the transfer of autologous DCs in combination with NAC-AC recovers the functionality of T cells. This was evidenced by the detection of p-AKT and p-ZAP70 upon the stimulation of their TCRs only in T cells from patients who received DCs.

### Tumor Infiltrating Lymphocytes’ TCR-CDR3 Sequence Increased in Peripheral Blood Cells After NAC plus DC Treatment

**Figure 6:**
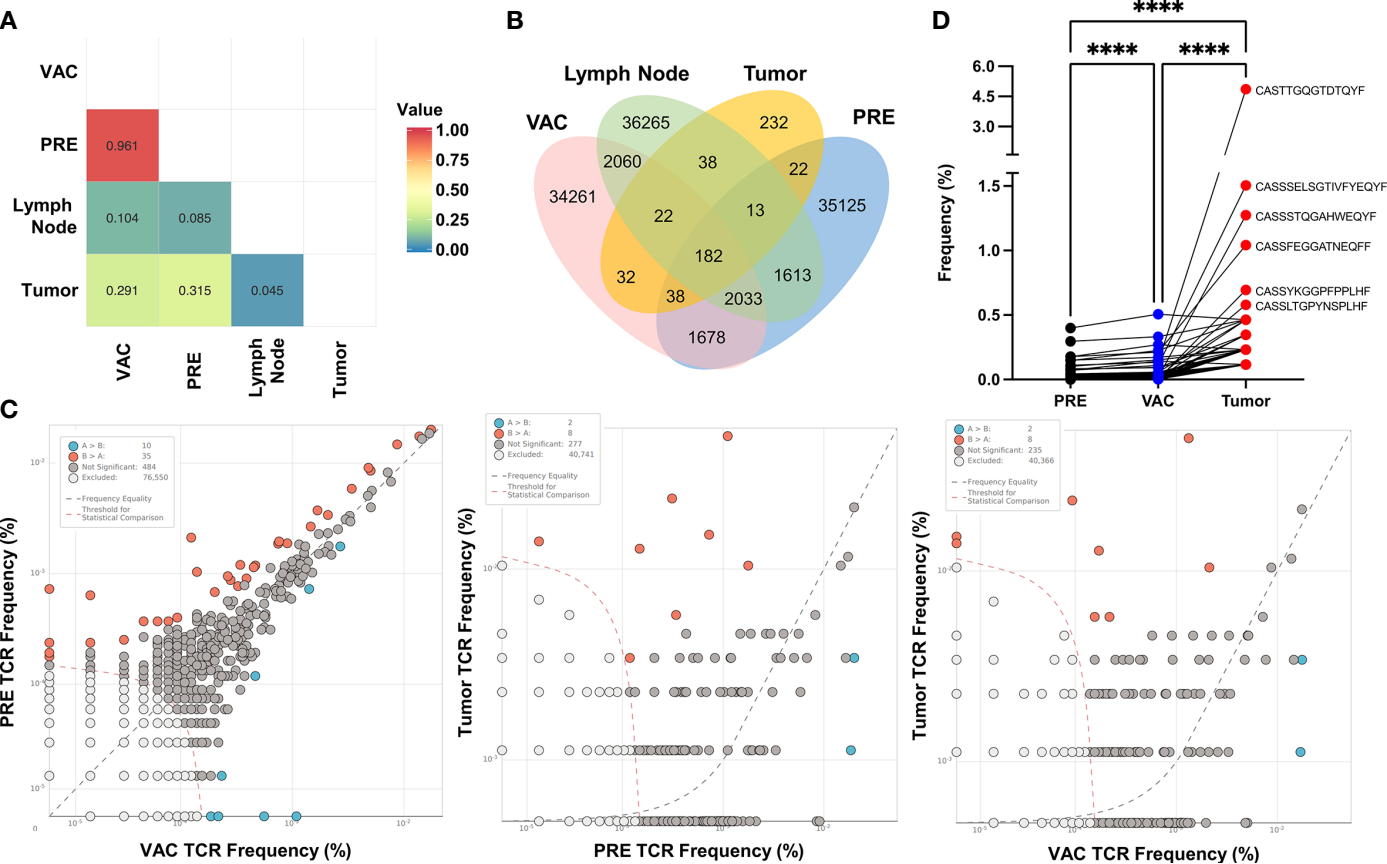
CDR3 sequences found in tumor-infiltrating lymphocytes correlates with expansion of T cells in a patient after doxorubicin and cyclophosphamide (NAC-AC) plus dendritic cell (DC) treatment. **(A)** Similarity analysis of the CDR3 sequence by Morisita index 0–1 (no similarity to complete similarity, respectively) represented in a heat map. **(B)** Venn diagram showing the number of overlapping rearrangements present in the four sequenced samples [tumor, lymph node, and peripheral blood mononuclear cells (PBMCs) before and after NAC+DC vaccination]. **(C)** Scatter dot plot comparing the frequency of unique sequences with significant differences (increased or decreased) between samples (orange and blue dots), excluded sequences [white dots, below threshold (orange dashed line)] and nonsignificant differences [gray dots, near frequency equality (black dashed line)]. **(D)** Paired comparison of the frequency of shared rearrangements among the three samples (n = 182), PRE (black dots), VAC (blue dots), and tumor (red dots). Top CDR3 sequences were denoted alongside the tumor CDR3 rearrangement. Parametric one-way ANOVA test with Turkey’s multiple comparison test among the three samples, ****p < 0.0001.

To assess if the immune response observed in the periphery of vaccinated patients could have a relationship with tumor-infiltrating lymphocytes (TILs), we determine the frequency and sequence of TCR-CDR3 region from PBMCs before and after vaccination (PRE and VAC, respectively) and compare them with the sequence of TCR-CDR3 detected in TILs from the remaining tumor and draining lymph node obtained during surgery from one vaccinated patient after NAC-AC in combination with DC transfer (VAC) using ImmunoSEQ service (Adaptive Biotechnologies) (**Table S1**). Initially, we determine the similarity between samples using a Morisita–Horn similarity index represented in a heat map (**Figure 6A**). As expected, PBMCs from PRE and VAC samples were the most similar (0.961) and very dissimilar among PBMCs compared to the tumor or lymph node cells with a slight increase in similarity between lymph node with VAC sample compared to PRE (0.104 *vs.* 0.085, respectively).

Next, we evaluate the number of overlapping sequences found in each sample using a Venn diagram (**Figure 6B**). Notably, there were more sequences shared between tumor and VAC samples compared to the tumor and PRE samples. To evaluate any significant differences in the frequency of CDR3 among samples, we compare two samples in a scatter dot plot to identify the rearrangements (unique sequences) that have significantly increased or decreased their frequency between the samples (**Figure 6C**). In the first plot (left), the majority of the sequences were excluded (by the low frequency) or were nonsignificant by their similar frequency found in both samples, although some rearrangements significantly increased in PRE (orange dots, n = 35) and others increased in VAC (blue dots, n = 10). This result is in concordance with the similarity between PRE and VAC samples. When comparing the rearrangements of the TILs *vs.* PRE or VAC samples (**Figure 6C**, middle and right plots), we found the same number of sequences that differs from PBMCs. Finally, we compare the frequency of the shared rearrangements in PRE, VAC, and tumor TCR-CDR3 sequences using paired analysis (**Figure 6D**), the majority of the TCR-CDR3 sequences found in TILs have significantly increased frequency after NAC-AC plus DC (VAC) compared with the PRE sample. These results suggest that, in this patient, the combined treatment increased the frequency of T cells in blood that have the same TCR-CDR3 that were found inside the tumor (TILs).

## Discussion

Current development in immunotherapy against cancer has shown great advances with the results of antibodies against checkpoints for non-small cell lung cancer and melanoma and with the use of cellular modifications such as CAR-T for B (CD19+) lymphoid leukemia. However, in pathologies such as breast cancer and many other solid tumors, advances in immunotherapy are incipient. One immunotherapy scheme evaluated in BCPs has been the use of DCs in different combinations of antigens and routes (15, 23). The safety of using adoptive transfer of monocyte-derived DCs has been proven in multiple clinical studies in different types of solid tumors (24, 25). Despite its safety and some cases of evident clinical response, the use of DCs has been focused as monotherapy in patients in advanced stages of the disease and dependent on the use of these cells pulsed with tumor-specific antigens, the latter greatly limiting its massive use given the restriction of antigens defined for therapy and the limited selection of antigens that bind to the HLA of patients. Concerning this point, recent advances are focused on the determination of personalized tumor neoantigens. Although this principle makes them applicable to any patient, the process of identification, validation, and synthesis of the neoantigen for each patient makes this option a complex system and currently expensive. Our approach in this clinical study in patients with infiltrating breast cancer was to take advantage of the environment generated by NAC, which from our studies and others is known to generate immunogenic cell death favoring recovery of T-cell functionality and maturation of DCs (10, 21, 26). Immunogenic cell death opens the possibility of exploring the antigenicity of tumors as cryptic vaccines and as a source of tumor antigens in cancer patients during their conventional treatment with chemotherapy (27–29) generating an environment conducive to the adoptive transfer of DCs derived from monocytes, allowing the possibility of transferring them without an exogenous antigen, since this is provided by the same tumor that is being treated with NAC. This novel approach, if effective, would eliminate the need for exogenous use of tumor antigens, which are expensive to obtain or synthesize to be clinically applicable. Additionally, the monocyte-derived DCs that were used in this study takes only 48 h of culture, which reduces the costs in time, media, and cytokines necessary for the generation of doses compared to the standard protocols of DCs derived in 7 days.

As this is the first clinical study in Colombia with DCs derived from monocytes, we evaluated the safety and immunogenicity of these cells in patients newly diagnosed with infiltrating ductal breast cancer who were treated with NAC for a minimum of three cycles. DCs were derived from autologous monocytes with two previously defined maturation cocktails (14, 30, 31). The mature DCs were dosed interspersed with the NAC-AC scheme in areas close to the tumor, with the aim that these cells were capable of capturing tumor antigens. Following what has been reported by multiple research groups in the world so far, the use of DCs is a safe immunotherapy system; none of the vaccinated patients presented a moderate or serious adverse event. Notably, we found a mild adverse effect in the majority of patients characterized by a reddening of the DCs inoculation area between doses 5 and 6, possibly due to the presence of IL-12 produced by alpha DCs used in the last doses (30); however, this effect was resolved spontaneously 24–36 h later without requiring treatment. We are currently in the clinical follow-up phase to determine favorable clinical response in patients who received DCs concomitantly with chemotherapy.

With the immunomonitoring process carried out on patient samples previously established by us (10), it was possible to determine the functional capacity of T cells before and after treatment. Notably, we observed in addition to the deficiency in CD3 internalization in patients before treatment differences between the two treatment regimens, chemotherapy alone and chemotherapy in combination with the transfer of DCs. In the first place, we observed that despite the internalization of CD3 measured by the decrease in MFI on the cell surface, it is associated with an increase in CD3 MFI at the cytoplasmic level, but not with TCR levels (data not shown), possibly explained by recirculation or degradation mechanisms that are independent between CD3 and TCR chains. Expression of CD69 reflects an early phase of TCR/CD3 stimulation (32) and may be related as a metabolic gatekeeper for most of the lymphocyte and NK functions (33) involving mTOR and STAT3 signal pathways. Most of the T cells after *in vitro* stimulation obtained from patients after vaccination with DCs express CD69 in cells that internalized the TCR/CD3 complex (**Figure 2D**). This result suggests that the combination of A/C chemotherapy with autologous DC transfer may help to recover an early activation profile of lymphocytes including T cells associated with TCR/CD3 activation and could be reflected in the downstream signaling pathway.

On the other hand, when analyzing if the intracellular signaling was directly dependent on the stimulation of TCR by the phosphorylation of ZAP70, it was not possible to find significant changes between the groups analyzed in response to the *in vitro* stimulation of T cells in the different subpopulations. However, the increase of p-ZAP70 in CD4+ T cells with TCM phenotype was striking. In samples obtained from patients before their treatment, we observed a lower response to the *in vitro* stimulus compared to the other three groups where there was an increase of approximately 50%. In addition to the phosphorylation of ZAP70, we evaluated the AKT–mTOR pathway in response to *in vitro* stimulation. Importantly, we found that in CD4+ T cell TEF, phosphorylation of AKT was decreased in patients before and after treatment with NAC compared to the normal response of HDs; however, patients who received NAC-AC combined with DCs have levels equivalent to those of HDs. Finally, something similar was observed when quantifying the phosphorylation of mTOR in the different memory subpopulations, we found a decrease in p-mTOR levels in CD4+ T cells (both in TCM and TEF) and CD8+ T cells in TCM in patients after the treatment with NAC-AC; this is a known effect of doxorubicin therapy where the production of reactive oxygen species (ROS) activates the AMP-activated protein kinase (AMPK) pathway, and this activation leads to the inhibition of mTORC1 (34).

It is noteworthy that the memory subpopulation in the patients who received combined NAC therapy with the transfer of DCs restored the phosphorylation capacity of mTOR to levels similar to those of HDs and with levels significantly higher than those in patients treated only with NAC. Together, these results support that the combined NAC plus DCs therapy favorably affects the functionality of different subpopulations of T cells, resembling the response capacity of HDs. The role of the interaction of immunotherapy with DCs and the responsiveness of the mTORC1 and 2 complexes in T lymphocytes is still unclear, where it has been shown that the use of rapamycin regulates the differentiation of T cells (35). However, the control of the metabolism of DCs is of great importance for the capacity of capture and presentation of antigens (36). Several clinical studies have been focused on the modulation of phosphoinositide 3-kinase (PI3K)/Akt/mTOR pathway to overcome chemotherapy or hormonotherapy resistance in breast tumors such as everolimus (37) or temsirolimus (38); this intricated pathway is not yet fully understood and need more studies (39).

New multiparametric analysis technologies such as CyTOF allow deep characterization of flow cytometry with more than 50 parameters (40). Initial automated processing systems assumed data behavior as positive or negative and not as an expression gradient (41), limiting the sensitivity of the analysis to detect rare populations. Current algorithms, such as SPADE (42), FLOCK (43), viSNE (44), ACCENSE (45), and more recently CITRUS (19), have provided the necessary tools for automated multidimensional analysis of these data to facilitate the interpretation and analysis of these complex phenotypes among the analyzed samples. Certain tools, such as CITRUS, are designed not only to define biological markers that differentiate the analysis groups but also to establish to which group an unknown sample could belong. These tools have been used mainly for the analysis of the cellular hierarchy in bone marrow samples from patients with leukemia and some in response to cellular mechanisms after drug treatment (46). However, to date, there is no published evidence that its use has been reported as a tool for immune monitoring of the clinical response to neoadjuvant chemotherapy in BCPs.

When we analyzed our immunomonitoring results using FlowSOM and CITRUS, we mainly found two cell groups that are significantly different between patients and HDs. In the first place, a population of CD8+ T lymphocytes with virgin phenotype with a certain degree of activation, defined by high relative levels of CD69 and CD25 (**Figure 5B**) that are increased in patients who received NAC plus DCs (VAC) compared to patients prior to treatment and mainly to patients treated but who did not receive autologous DCs. Finally, when intracellular signaling markers were expanded, we observed a similar behavior of CD8+ T cells of naive phenotype with high levels of phosphorylated ZAP70 and AKT (**Figure 5E**; cluster 178097), associated with this result, a second cluster (**Figure 5E**; cluster 178119) has a phenotype characterized by low levels of CD3, TCR, CD45RO, and CD8, also with high levels of CD62-L, p-ZAP70, and p-AKT, which would be compatible with T cells in a higher state of activation probably by the internalization of the TCR complex and due to high levels of phosphorylation by ZAP70 and AKT. This type of population could belong to memory stem T cells (TSCM) (47); however, it is necessary to characterize these cells with markers that have been described for TSCM such as CD95.

Finally, we propose that the evaluation of the T-cell repertoire by sequencing the CDR3, present in blood and tumor after vaccination of one patient, could lead to identifying and correlating the frequency of T cells that infiltrate the tumor and the behavior of these cells in the periphery after treatment. We found a higher frequency of the shared rearrangements in the PBMCs after the vaccination compared to before vaccination related with the sequences found in the tumor (**Figure 6D**). These results suggest that the recovery of immune response detected in these patients’ PBMCs could be associated with specific T-cell expansion after treatment with NAC-AC plus DCs with the same TCR of TILs (**Table S1**) as previously suggested by our group (9). Including the analysis of TCR-CDR3 sequence in clinical trials like this could be the standard technique for immunomonitoring the repertoire of T cells and their response to cell transfer as a vaccine or as evaluated by others using neoantigens and anti-PD-1 treatment in melanoma patients (48). However, these results must be validated in a larger cohort of patients, taking into account that the majority of tumors disappear after NAC-AC, to obtain a representative sample of the infiltration of T lymphocytes in the tumor microenvironment and its association with the response in peripheral blood cells and should be analyzed in patients who received standard treatment (NAC) compared to patients who received NAC-AC plus DCs.

In conclusion, this clinical study confirms the safety of monocyte-derived autologous DC transfer in 2 days in combination with primary chemotherapy with doxorubicin and cyclophosphamide. DCs would take advantage of the induction of immunogenic cell death generated by this chemotherapy scheme to generate a greater response of T lymphocytes, where we observed that in the patients who received the combined therapy, the ability of the T cells to respond to an unintended stimulus *in vitro* was better compared to patients who did not receive DCs. Follow-up of these patients should continue to determine whether these immunological effects have a long-term impact on survival and to determine the mechanisms involved in the restoration of the immune response of DCs and T cells in patients with this type of chemotherapies. This type of study would open the possibility of combining the transfer of DCs with the use of anti-checkpoint antibodies such as PD-1 or programmed cell death ligand 1 (PDL-1), which could favor the antitumor immune response.

## Data Availability Statement

The datasets presented in this article are not readily available because they are not genomic or sequence data. Requests to access the datasets should be directed to caparral@unal.edu.co.

## Ethics Statement

The studies involving human participants were reviewed and approved by Comité de Ética e Investigaciones de la Universidad del Rosario, Hospital Universitario Mayor de Méderi. The patients/participants provided their written informed consent to participate in this study.

## Author Contributions

DB-E and CP-L: wrote the paper. DB-E and MO: executed the experiments. DB-E, MO, PO-M, and CP-L: analyzed the data. DB-E, CC, RS, and CP-L: patient monitoring and clinical assessment. All authors contributed to the article and approved the submitted version.

## Funding

This work has been supported by a joint grant between Fundación Salud de los Andes and the Universidad Nacional de Colombia, DIB, Vicedecanatura de Investigación Universidad Nacional Medical School (HERMES: 42207 and 41790) and MinCiencias/COLCIENCIAS (grant 110150227509 CT. 671-2013 and grant 6952080763382 CT. 840-2018).

## Conflict of Interest

The authors declare that the research was conducted in the absence of any commercial or financial relationships that could be construed as a potential conflict of interest.

## Publisher’s Note

All claims expressed in this article are solely those of the authors and do not necessarily represent those of their affiliated organizations, or those of the publisher, the editors and the reviewers. Any product that may be evaluated in this article, or claim that may be made by its manufacturer, is not guaranteed or endorsed by the publisher.

## Supplementary Material

Supplementary Figure 1 Schematic model of multivariate analysis of flow cytometry data. Workflow for the analysis of flow cytometry data from acquisition (file in FCS format) and manual analysis. For automated analysis concatenated samples were dimensionality reduced using tSNE. Cluster generation heat maps and hierarchical tree were done using FlowSOM plugin in FlowJo (BD). For CITRUS analysis implemented in R software, FCS files were exported from live singlet cells in FlowJo and then executed using CITRUS command. Data obtained was analyzed in Prism (v9) for statistical confirmation.

Supplementary Figure 2 Validation of FlowSOM and CITRUS in a control group. **(A)** Analysis of PBMCs obtained from HD (n=10), stimulated or not during 24 h with beads coupled with anti-CD3, CD28 and CD2. PBMCs were labeled with antibodies against CD3, CD25, CD69 and CD154. tSNE and density plots of concatenated samples overlaying one stimulated sample (Red) and one unstimulated sample (blue). **(B)** FlowSOM results from 10 HD samples to determine tSNE map and eight sub-populations. **(C)** Spanning tree of the eight sub-populations defined by FlowSOM showing the expression level of each marker (pie chart). **(D)** Heat map and hierarchical distribution of the populations determined by FlowSOM, and **(E)** distribution of each population in control or stimulated cells (blue and red bars respectively). **(F)** CITRUS analysis of the populations represented in the spanning tree. **(G)** Expression levels of each marker (red histogram) compared to background (blue histogram) in each cluster whose expression exhibited statistical difference between both groups (unstimulated *vs.* stimulated cells). **(H)** The model curve of CITRUS for false Discovery rate (blue line) and cross-validation error rate (red line). **(I)** The relative abundance of three clustered cells that exhibited statistically different patterns of CD3 internalization in cells of 10 HD unstimulated or stimulated with beads *in-vitro* is presented as control and stimulated respectively.
