# Supplementary figures and images for: Autologous Dendritic Cells in Combination With Chemotherapy Restore Responsiveness of T Cells in Breast Cancer Patients: A Single-Arm Phase I/II Trial
Source: Front Immunol. 2021 Aug 20;12:669965. doi: 10.3389/fimmu.2021.669965 (PMC8417880; doi:10.3389/fimmu.2021.669965)

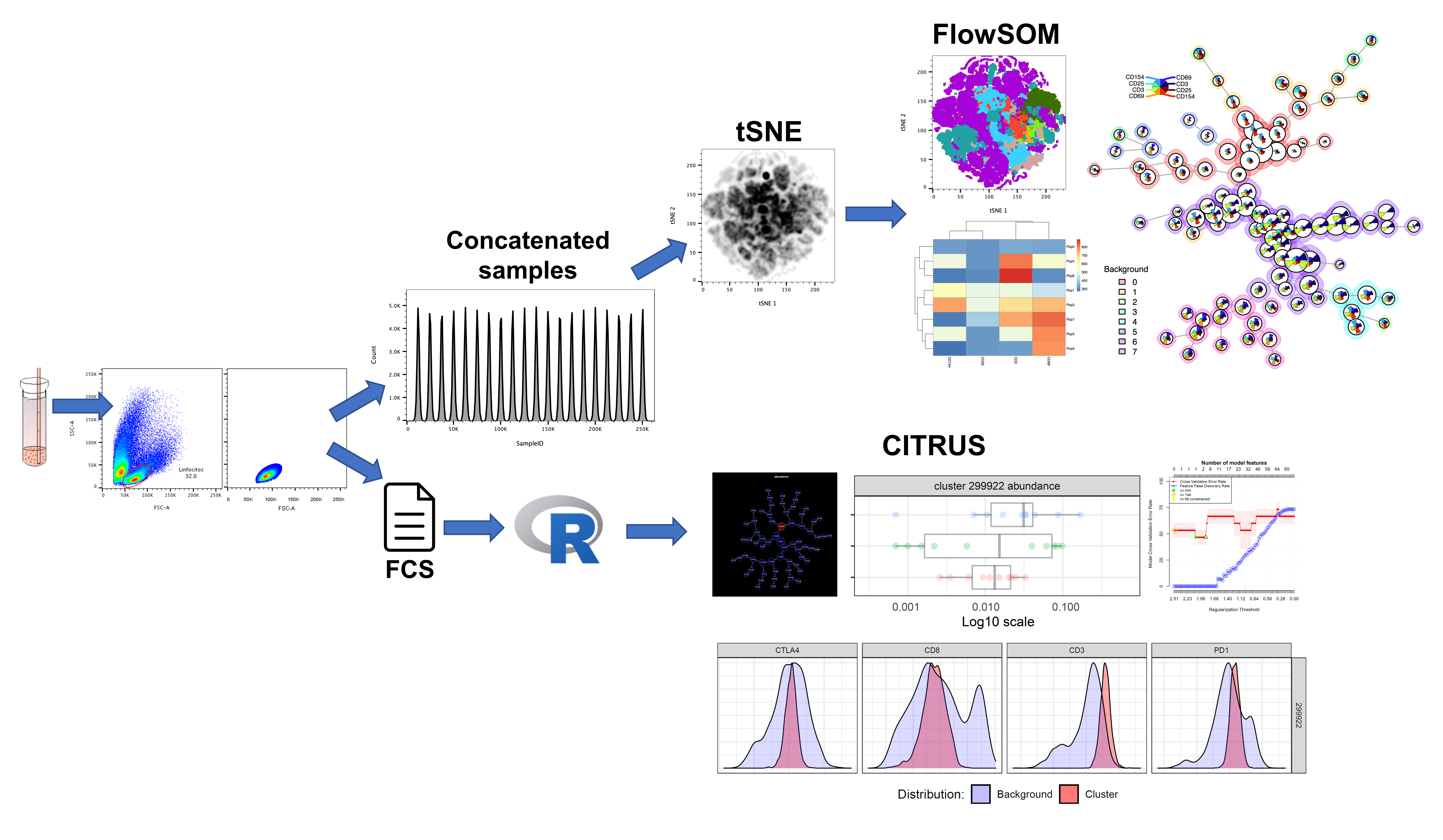

Supplement: Supplementary Figure 1 — Schematic model of multivariate analysis of flow cytometry data. Workflow for the analysis of flow cytometry data from acquisition (file in FCS format) and manual analysis. For automated analysis concatenated samples were dimensionality reduced using tSNE. Cluster generation heat maps and hierarchical tree were done using FlowSOM plugin in FlowJo (BD). For CITRUS analysis implemented in R software, FCS files were exported from live singlet cells in FlowJo and then executed using CITRUS command. Data obtained was analyzed in Prism (v9) for statistical confirmation. [file Image_1.tif]
